# Navigating misinformation and political polarization of COVID-19: interviews with Milwaukee, Wisconsin county public health officials

**DOI:** 10.3389/fpubh.2023.1215367

**Published:** 2023-12-21

**Authors:** Garrett Bates, Mohammad Titi, Julia Dickson-Gomez, Staci Young, Aliyah Keval, John Meurer

**Affiliations:** ^1^Institute for Health and Equity, Medical College of Wisconsin, Milwaukee, WI, United States; ^2^School of Medicine, Medical College of Wisconsin, Milwaukee, WI, United States; ^3^Department of Family and Community Medicine, Medical College of Wisconsin, Milwaukee, WI, United States

**Keywords:** COVID-19, vaccine, public health leadership, political polarization, public health burnout, risk severity, COVID-19 misinformation

## Abstract

**Introduction:**

The spread of misinformation combined with the political polarization of the COVID-19 vaccine created major challenges for public health officials responding to the COVID pandemic and vaccine roll-out. The challenges public health officials faced when making safety recommendations and promoting the vaccine only exacerbated the already exhausting work conditions they experienced since the start of the pandemic. Combating misinformation while receiving inadequate political support led to burnout for many public health officials. As such, they had to adapt and develop new strategies for increasing vaccine acceptance and decreasing vaccine hesitancies.

**Method:**

This study was conducted through qualitative interviews with seven Milwaukee County public health officials. This study aimed to determine how public health officials perceived misinformation and political polarization during the pandemic. Additionally, the study aimed to learn more about strategies county health officials used to combat misinformation while increasing vaccine uptake in their communities.

**Results:**

Thematic analysis of the interviews identified three major challenges faced by public health officials in promoting vaccination: dissemination of misinformation in media, political polarization of COVID and its contribution to vaccine acceptance and COVID fatigue, and assessment of the risks associated with disease severity versus vaccine safety considering limited public health resources.

**Discussion:**

Learning from public health officials allows us to better understand their perceptions of the extent of local vaccine hesitancies and their advice on how to counteract fears and misinformation and to promote COVID vaccine uptake. Political polarization of COVID and misinformation affected community vaccine acceptance and challenged local public health leadership.

## Introduction

The COVID vaccine has been proven to be safe and effective through rigorous efficacy trials. Vaccine effectiveness monitoring found the odds of hospitalization fell by about 70% after one or two doses, the chances of severe disease (having five or more symptoms in the first week of illness) dropped by about one-third, and the likelihood of having long COVID (symptoms for at least 28 days after infection) was halved (1). The addition of COVID boosters has increased overall vaccine effectiveness in protection against infection to nearly 70% among adults and 94% effectiveness in preventing death (2). However, despite considerable evidence of safety and effectiveness, vaccine uptake has been suboptimal.

## Literature review about misinformation

### COVID-19 vaccine

Vaccine safety is a consistent concern for many people and can be a primary factor in their hesitancy to receive the vaccine. Research has found that people who believe vaccines are unsafe are less willing to receive them, know less about the infection, and are more likely to believe misinformation about the vaccine (3). Additionally, research findings suggest that those who believe the COVID vaccine to be unsafe have lower levels of health literacy, less formal education, lower income, and are more likely to live in rural areas than people who believe the vaccine is safe (4). In Milwaukee County, 90% of residents aged 25 and over have a high school diploma or higher, while only 32% have a bachelor’s degree or higher (5).

Research suggests that communities of color tend to have low vaccine confidence and high vaccine hesitancy (6, 7). For historically marginalized groups, such as African Americans, their history of oppression when seeking adequate healthcare, and persistence of significant health disparities in the present, make it even more challenging to overcome hesitancy for a new vaccine, like the one for COVID (6, 7). Research suggests vaccine acceptance can be increased and uncertainty reduced more effectively through vaccine interventions, whereas removing the choice through a mandate may negatively impact vaccine acceptance (8).

The COVID vaccine has been met with much pushback and hesitancy since its initial roll-out. As of February 2023, 62% of Milwaukee County residents have completed the primary COVID vaccine series, well below the United States average of 69% (9, 10). Vaccination rates are highest among people above age 65, females and Asians and lowest among youth, men, black individuals, and Hispanics (10). Of all Milwaukee County residents, only 15% of the population have received the bivalent booster as of February 2023 (10). Only 17% of Wisconsin residents received the current COVID booster, roughly the same as the 16% US average (2, 9). These low rates of vaccine uptake could result in a resurgence of COVID cases, hospitalizations, and deaths. It is essential to increase vaccinations and boosters. Evidence suggests the leading way to increase vaccine acceptance and uptake is through intervention projects that reduce vaccine hesitancies and promote accurate information by boosting confidence in the safety and effectiveness of the COVID vaccines, combating complacency about the pandemic, and increasing the convenience of getting vaccinated (11).

### Media

Research findings indicate people have concerns about side effects and safety of the COVID vaccine, lack trust in the government, and are concerned that COVID vaccines were developed too quickly (12). However, unlike past vaccines, the decision to receive the COVID vaccine is also heavily affected by cultural norms, social and peer influences, and political views (6). Distrust of the government and health care systems has contributed to COVID vaccine hesitancies for many Americans (6). Opposition to the COVID vaccine by media outlets, political polarization of COVID, and the spread of misinformation has further reduced vaccine acceptance, especially in lower-income areas of the US (7).

### Political polarization

The bi-partisan structuring of the US is believed to have contributed to political polarization of the COVID pandemic as people received information from polarizing, biased informational sources while having decreased cross-partisan social interactions and information sharing (13). Much of public health response during the pandemic, including safety recommendations, social distancing, mask wearing, and vaccine promotion, was disseminated through various media outlets. US media sources with differing political alignments portrayed COVID differently; certain politically charged media sources reported more negatively about COVID and recommendations made by health authorities (13). Throughout the pandemic, decision making authority was questioned as political parties were divided on how to respond to the pandemic while considering how the US economy would be affected, further influencing the polarization of public opinion (14). More research is needed to better understand how political polarization can be mitigated so that it does not affect public opinion to the degree it has throughout the COVID pandemic.

### Public health leadership

COVID fatigue is a growing problem for the general population and the healthcare system as the pandemic lingers. A COVID-fatigued population in combination with health care provider burnout has exacerbated an already stressed health care system. Since 2020, 1 in 5 healthcare workers have quit their jobs, and over 50% of those who quit cited the COVID pandemic and burnout from work as main reasons (15). Burnout among healthcare providers increased as COVID related hospitalizations increased, many of which could have been prevented by increasing COVID vaccination rates, especially with the bivalent booster. More specifically, public health workers, compared to healthcare providers, saw even greater levels of burnout during the pandemic, accompanied with reports of exhaustion, anxiety, and depression (16). Over two-thirds of public health officials have reported experiencing increased burnout, many of whom also reported experiences of professional abuse, harassment, and personal threats which negatively impacted their jobs, further increasing burnout (16, 17). For many public health workers, the burnout, harassment, stress, and depression stemming from the pandemic proved to be too much which has led to the resignation of hundreds of US public health officials since 2020 (17). Further research is needed to better understand why public health officials experienced such high levels of burnout, what was done to alleviate that burnout, and additional negative impacts they experienced while performing their duties to serve and protect their communities.

### Study objectives

Factors affecting vaccine acceptance among Milwaukee County residents during the initial roll-out of the COVID-19 vaccine challenged public health officials who responded with new strategies. Public health officials provided their observations and experiences of factors that influence community beliefs about health interventions. Study findings can be used to counteract misinformation and to support public health officials during the next public health crisis. This study provides new insight, and a better understanding of how public health officials were constantly challenged by rapid, vast dissemination of misinformation and unsupported by decision makers. The challenges health officials faced led them to feel overwhelmingly burned-out and that they were no longer trusted as a key source for COVID prevention and safety information. The purpose of this study was to answer two major research questions using public health interviews:

1. How did misinformation and political polarization of COVID and the COVID-19 vaccine affect how Milwaukee County public health officials performed their duties and responsibilities during the pandemic?
2. What COVID vaccine confidence boosting strategies were used in Milwaukee County and what additional strategies were used by public health officials to counter misinformation and increase vaccine uptake among the different communities in Milwaukee County?

## Methods

### Study setting

Milwaukee County is one of the most racially and economically diverse and segregated counties in Wisconsin; it is home to approximately 920,000 adults with 28% Black or African American, 16% Hispanic/Latinx, 5% Asian, 1% American Indian and Alaskan Native, and 50% White/Non-Hispanic in 2022 (5). Low income and poverty are challenges faced by many Milwaukee County residents. The percent of persons in poverty in Milwaukee County (18%) is almost double the whole state of Wisconsin and the median household income is $55,000, compared to $67,000 for the state (US Census, 2021). Almost 10% of residents do not have health insurance, compared to almost 7% for the whole state (US Census, 2021).

### Study design

The study was performed using an exploratory approach through qualitative interviews with seven public health officials in Milwaukee County. An explanatory design allowed for construction of interview questions that would obtain in depth and diverse responses from public health officials (18). No one health official’s response to a question was the same as another’s responses. Interviews with local public health officials allowed an analysis of unanticipated comments and to better understand responses in real-time, allowing the interviewer to ask additional follow-up questions (19). Interview questions were guided by findings from our literature review, the specific aims of the study, and the results of focus group interviews with Milwaukee County residents regarding COVID risks that were conducted earlier in the study (20). Interview questions asked about the vaccine trends public health officials witnessed, factors they noticed contributing to vaccine acceptance in their communities, vaccine promotion ideas, and interventions that they conducted to increase vaccinations among at risk and hesitant populations. Participants were provided the interview guide in advance so that they could prepare accordingly and share as much information as possible. Participants were given the opportunity to email study staff with any questions regarding the interview or study protocol.

### Recruitment

Our goal was to establish a heterogeneous group of public health officials from various jurisdictions of Milwaukee County. The principal investigator emailed Milwaukee County local health department leaders to recruit them to the study. The Medical College of Wisconsin Human Rights Review Board reviewed and approved all study activities. Participants were sent an informed consent informational letter prior to the interview. Participants verbally provided informed consent upon their involvement in this study and were informed of additional research outcomes that may stem from their participation. Seven public health professionals were interviewed between March 30 and May 18, 2022. Five were women, two men. Their educational credentials included MPH (4), MS, MA, RN, and MD. Their titles included health officer (5), director (2), and nursing supervisor. They worked at city health departments (6) or a county health department.

### Data collection

Interviews were conducted by Zoom and lasted approximately 30–45 min each. Interviews were professionally transcribed verbatim and deidentified for analysis.

### Interview protocols

Public health officials responded to interview questions addressing three different survey constructs: social media activity, COVID and perception of risk, and public health employee burnout. An example of each construct is listed below. The interviewer addressed each area as thoroughly as possible in the 30–45-min span allotted for the interviews. Public health officials were open with their responses and provided detailed responses to each question and any follow-up questions.

- Social media activity: What forms of media, do you think, have been accurately communicating ‘the facts’ to the public? (e.g., specific TV, print news, radio/podcasts).
- COVID and perceptions and risk: Do you have any concerns about COVID vaccines, or the way in which they are being used? (e.g., use in adults vs. use in children). How can those concerns be reduced?
- Public health employee burnout: What are some of the reasons you have noticed that have led to public health officials transitioning out of their field?

### Data analysis

An inductive analysis approach was used which included open coding, creation of categories, and abstraction (21). Our research team read the transcribed interviews multiple times to understand the shared information. A coding tree was created to capture specific terms or phrases using an inductive coding approach in which codes were generated as the transcribed interviews were read and analyzed. Once all text segments were coded, we then created categories and further synthesized into themes. Meaning was given to codes through the categorization process. For instance, specific codes were assigned to text segments that mentioned vaccine hesitancies, vaccination strategies, vaccine misinformation, etc., but these coded segments were all then categorized as “Contributing factors for vaccination.” Intersecting codes and coded segments were identified which allowed for the recognition of relationships and theme development. Direct quotes and phrases from public health officials were analyzed for further meaning which led to the generation of possible themes, as part of the contextualization process. Themes were further developed through abstraction, using reoccurring codes and contextualizing quotes from interview participants. MAXQDA software was used for coding and generating reports with coded segments and quotes to be used for analysis. Noteworthy text segments were highlighted and used to support the credibility of our themes. Quotes were selected to be included in the results section to follow. Descriptions were developed from the reoccurring themes which provided further context to further support the created themes discussed throughout the results.

## Results

**Figure 1:**
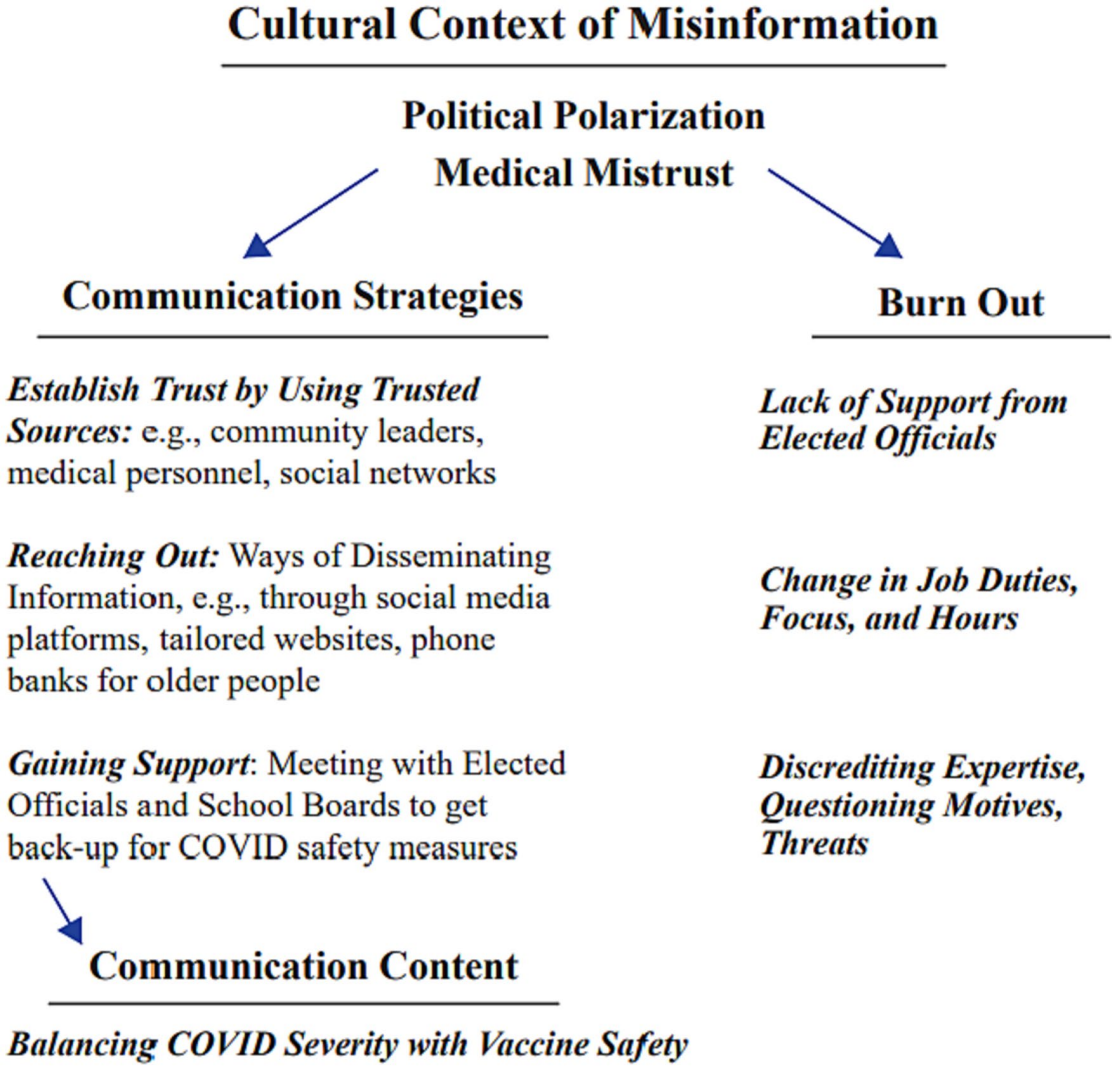
Strategies used by public health officials to navigate the effects of misinformation, medical mistrusts, and political polarization during the pandemic are included in the flow chart above to the left. The resulting challenges health officials faced (burnout) during the pandemic are included on the right. Together the two columns depict the continuous efforts public health officials made while serving their communities throughout the pandemic.

Using thematic analysis, we identified the following three core themes: (1) misinformation in the media; (2) the role of political polarization in COVID fatigue; and (3) weighing the risks of COVID severity vs. vaccine resources. Descriptions of communication strategies public health officials used to counter-act misinformation and disseminate accurate information are included in a flowchart at the conclusion of the results (Figure 1). The flowchart additionally includes challenges public health officials had to persevere as they fulfilled their duties and responsibilities during the pandemic.

### Misinformation in the media

When asked about how the population they served learned of the COVID pandemic and vaccine rollout updates, public health officials responded that they received information from various news outlets including television, radio, internet websites, and social media. Different media outlets delivered their messages in different ways and not all messages contained accurate information. Participants reported that it was difficult to monitor news delivered through social media for accuracy.

There were multiple instances where myths and facts regarding the COVID vaccine were mixed. All participants reported how social media allows for the dissemination of misinformation. One stated:

*“I think one of the themes throughout the pandemic given how politicized, for better or worse, that the topic of COVID became, was really coming to the reality that social media in particular is a platform that can promote misinformation, disinformation, or accurate information.”*

Another noted how they *“felt and experienced the platform of social media providing both disinformation and misinformation.”*

Multiple health officials mentioned vaccine safety was a concern among adult community members, even noting rumors circulating about the vaccine being unsafe or that it contained “trackers”:

*“When the vaccine became available, especially early on, we had quite a few questions about the safety, rumors about trackers or those things are in vaccines.”*

One participant noted they came across a social media post saying, *“Vaccines Kill”* followed by a several comments with some people in agreement. Social media was not the only source of COVID misinformation. Participants also mentioned that community members received information from radio or television news programs addressing the COVID vaccine in negative ways and that these sources also sometimes disseminated false claims about the vaccine and severity of COVID. With so many sources of information available to community members, health officials took on the responsibility to monitor the public’s perception of the COVID vaccine.

Misinformation about disease severity and mortality caused people to question their need to be vaccinated. Another health official mentioned they had community members denying that deaths attributed to COVID were caused by COVID, claiming that the deaths were caused by other factors:

*“They were saying that we are exaggerating the seriousness of COVID, or the impact that it’s having on some individuals, or the number of individuals that are dying. Thinking that the death data is being exaggerated or the – you know, we have those conversations with some stories, ‘Oh, they died in a car accident, but they had COVID, so you said they died of COVID’.”*

Claims that COVID mortality rates were much lower than those officially reported were common in those communities. No evidence was provided to support these claims, but they circulated, nonetheless.

Combatting misinformation is a challenge in the public health sector and necessary for increasing COVID vaccine acceptance. When asked how they responded to misinformation while increasing accurate information, our health officials provided several different strategies, such as working to obtain community leader support for their decision making on COVID recommendations and empowering grassroots community movements. When investigating strategies to connect with community members, one health official noted:

*“As we look over the span of the pandemic to date, we really just want to recognize when we empowered grassroots community neighborhood members to amplify messages on their own social media platforms, Facebook, TikTok, you name it, whatever, that is where we really were seeing some of the most direct influence to some of the most vulnerable populations and had the best sort of reach.”*

Multiple public health officials noted the importance of connecting with their community members during the complicated times of the pandemic, the continued use of grassroot campaigns when developing effective messaging, and the collective efforts of public health and community centers when promoting accurate information. These public health officials took charge and noted how it was their responsibility to disseminate accurate information to their communities. One health official explained their unbranded, custom designed strategy for promoting truth about COVID information and testing through development of a dedicated, COVID informational website to be used by all community health organizations and unrestricted by a sole health entity:

*“As the pandemic marched forward and time passed, that then got converted into healthy MKE. And so our kind of constant narrative was, “Come here for a source of truth about all things COVID.” And so that platform was created, again, with input from grassroots community members who were very clear about naming, “I want to see people from my neighborhood, who look like me, who are from my neighborhood and look like me that are behind the photographs that are on the websites. Who’s behind the camera matters, who’s on the webpage matters.” I think what was really exceptional about the work that we continue to do, it’s not branded. So, all of the health systems, all of the community health centers, other partners, contribute and used this. And we were able to, collectively, in the absence of a brand come together and say, “This will be our source of truth, to help streamline some of the narrative.”*

Another public health official mentioned how they tried to establish networks throughout their community so their decision-making regarding COVID safety recommendations would be supported:

*“With our schools, with our elected officials here, with our common council, and our mayor, and our administration. Spending time at those meetings, sending regular updates, often by email earlier in the pandemic, or having phone calls with them to answer their questions and help them understand. Also, a lot of messaging to different groups of businesses. So, you know, to churches, to childcare centers, to restaurants and bars, other businesses. So, really trying to send specific information that relates to those businesses or organizations as they were making decisions.”*

Several participants also noted using social media platforms to disperse accurate information and vaccine updates. Health officials had to adapt with the times and navigate social media usage and messages to the point where they had to “outcompete” possible sources of misinformation:

*“I think I would say we have a couple of our social media posts that you know kind of were shared widely, went viral, whatever you want to say. So, I do think that when we took the time to kind of make a higher quality graphic that would illustrate, whether it was data or mitigation strategy that we were recommending. I think that that was probably more of the most effective, just given the number of people who viewed it and shared it.”*

One public health department implemented a state funded survey that they disseminated to their community addressing health equity and barriers to vaccination with a focus on vaccine effectiveness and safety. They obtained over 500 responses. The survey participants had mixed opinions when asked what their trusted sources of COVID related information are. Some survey participants indicated public health and government officials as trusted sources, while other participants noted public health and government were not their first source for information, listing family and friends above government. The health department used results from the surveys, specifically the age range for participants who did note they use government and public health as sources of information, to tailor social media messaging and platforms. The tailored messages were perceived to be more useful for the targeted audience, residents in their 30s and 40s for the most part, who see the health department as a reliable source of information. Notably, the area this organization served had very high COVID vaccination rates.

Establishing trustworthy connections within the community was also recognized as a useful strategy for public health officials when addressing individuals’ concerns about the vaccine:

*“We realized the one-on-one support was much more likely to lead to somebody then getting the vaccine if they were able to talk to a nurse or talk to somebody and answer those questions by somebody they trust, that was actually a medical person.”*

One health official noted *“using CDC and DHS wording”* when recommending vaccines to their community members. Multiple participants stressed contacting older community members who may not have social media via phone calls or implementing “hotlines” (a 24/7 number that community members could call to ask vaccine related questions) to provide any vaccine updates and recommendations. Aforementioned strategies used by public health officials to navigate misinformation and promote accurate information are displayed in Figure 1 below. Ultimately their takeaways for effective strategies were developing vaccine intervention plans in different languages and tailored for different cultural groups and the re-direction and correction of misinformation public health officials came across on social media while trying to not be dragged into an argument or project any negativity.

### Political polarization and burnout

Effective vaccine roll-out was dependent upon politicians and public health officials working together to develop a vaccine dissemination plan that would boost local vaccine acceptance. However, with the vaccine being rolled-out during the conclusion of an election year in the US, health departments began receiving backlash and negativity as it became a hot topic in the political forum with continued lower acceptance by the Republican Party versus the Democratic Party (22). Overt politicization of the public health response, including widespread misinformation related to COVID vaccination, was spread by various forms of media and politicians (11). The public health officials interviewed through our study reflected on the high degree of opposition to the COVID vaccine leading up to and during the roll-out. The lack of political support for public health officials exacerbated already exhausting jobs. One health official detailed their need for support in their decision making from political or administrative leaders, while trying not to politicize the vaccine:

*“So, having leaders of the community also express their support, I think was impactful, for sure. And we have seen that for other type of public health responses as well. But honestly, though, it was a little bit harder for something like this because people did not really want to always get involved in something that’s controversial. Like, you know, “I support it, but I’m not gonna be public in supporting it.” I think that also got to be a challenge because it was so politicized.”*

With a country divided politically at the end of 2020, post-election, many Americans held fast to their beliefs. Participants in our study explained how more conservative media sources displayed more opposition to the vaccine than liberal media sources; thus, the more conservative community members who relied on those resources had more opposition to the vaccine. News outlets tend to be a source of information for many people; however, news stations do not always present a situation or event in the same way spreading contradictory information on a topic. FOX News and MSNBC, media sources traditionally on opposite ends of the political spectrum, were even discussed during one interview as being information resources for certain community members, creating further challenges for health officials attempting to combat misinformation.

*“Fox News was probably giving a much different spin than MSNBC on a* var*iety of topics. And so, I would say the place where that information originates, whether it’s with a local health department, a state health department, is probably more important than what channel it was on or what source of information was out there. It was very interesting to see all of the negative sources of information and disinformation or misinformation that came out.”*

Participants revealed how some community members and political leaders were in support of the vaccine, while others tended to downplay the severity of the pandemic and the need for a vaccine. One public health official explained their frustration by describing how their small team worked to provide services to their community while receiving pushback from “leaders”:

“*Eleven of us are working together in a very small geographic area with very fluid borders and irregular borders – being on the same page, supporting each other, providing prospective and experiences is gonna be very, very important. But that’s what we can do. I cannot change the political leaders in the village next door to me that basically chastised the health officer in an open public forum and said that what they are doing is unnecessary and inappropriate.”*

One public health official went so far as to say that vaccines were politicized to such a degree that for a Republican, getting vaccinated was tantamount to switching political loyalties.

*“I do not think in the history of public health have we ever, ever predicted something would be so politicized to this level, where it wasn’t really about really health, it was more political lines. You’re betraying a certain thing if you were doing it, to be honest, that was a lot of it.”*

Politicization of COVID not only affected vaccine acceptance, but also the day-to-day work of public health officials creating an already stressful work-life. Burnout due to COVID was encountered by all the public health officials involved in this study as they and their co-workers worked long hours, changed roles in the workplace frequently as co-workers retired or quit, and delivered difficult and important messages, recommendations, and restrictions to their communities. They fulfilled many different tasks and roles while receiving backlash from groups who opposed their guidance and working with a political system that sometimes failed to support them. When we asked our participating public health officials what exacerbated their feelings of burnout, they had many different responses, and all participants noted a lack of full support by a political system that they felt should be doing the most to unite and protect people:

*“We run up against a situation where politicians, political leaders, school leaders have pulled away mask mandates and mask guidance or even mask recommendations.”*

The same health official also discussed how the lack of support created additional burnout during an already overwhelming experience:

*“People got dramatically burned out. They got frustrated with the political process. They got frustrated with the community members who continue to chastise them on social media, print media, at public meetings. Discouraging or discrediting their expertise.”*

A second health official also noted the challenges politics created as they strived to fulfill their responsibilities and duties:

*“We’ve become a sort of lightning rod for threatening people’s freedoms and having a negative impact on the economy, when, in fact, all we were trying to do was save people’s lives. Like, at the end of the day that’s all any of us wanted to do but because of the politics related to the pandemic it’s become something very different.”*

Half of our participants shared how they or their coworkers had been personally attacked on social media or through their organization’s website. One health official stated they were sent a post with sheep wearing masks and were accused of committing crimes against humanity while trying to promote the COVID vaccine. Another participant shared how many health officials were threatened by various community members and elected officials while doing their job:

*“Burnout and exhaustion is probably the theme of it all amongst leaders. We were pretty fortunate here in our community where I can say I do not think our previous health officer and myself ever received death threats. I did not have to have police positioned outside my home. I did not have to be escorted to my car from board of health meetings or council meetings. But a lot of my peers did.”*

Participants explained how they had co-workers experience fatigue and burnout to the extent that they quit their jobs, further exacerbating burnout as workloads of those remaining increased. Some health officials quit due to the arduous nature of the work. Others found it to be a good time to change careers as they were forced to provide guidance and restrictions to people who viewed it negatively. Others had to change from working on a public health task they enjoyed to something they did not enjoy or felt they lacked the experience to do and opted out of the position as a result. While factors contributing to burnout varied for our public health officials; all health officials reported some level of burnout. A summary of challenges contributing to public health official burnout is included in Figure 1.

### Weight of COVID severity versus vaccine resources

Public health officials tried to help vaccine hesitant community members to weigh more appropriately the risks associated with the COVID vaccine with the risks of contracting COVID while health officials had to consider their own resource depth. Five public health officials reported that they encountered community members who were not concerned about contracting COVID because they perceived that they were at low risk for illness or minimized its severity. The severity of COVID proved to be a topic of debate along with the safety and effectiveness of the vaccine. One public health official explained how they had to weigh their limited resources and time when developing vaccine promotion messages. For some age groups, such as 65 and older, COVID complications can be more severe than in younger age groups. Additionally, parents who are already hesitant about childhood immunizations and fatigued from all the COVID information circulating already may be less receptive to public health messaging. Weighing resources for chronically underfunded public health organizations forced public health officials to make some difficult decisions. When deciding on how to use resources, health officials must address who can benefit most from public health messaging:

*“We’re weighing the risks and the benefits of continuing to talk about COVID vaccines specifically when we have seen such a decline in routine childhood immunizations. What are the risks and the benefits of folding COVID into that, or attaching it into that messaging?”*

Participants found promoting the COVID vaccine for children even more challenging, especially those with anti-vaccine parents. Children often have mild symptoms and parents assessed the vaccine as being a greater risk than COVID illness. While complications from the vaccine are extremely rare, they are not zero. One health official mentioned the risk of myocarditis in adolescent boys who received the vaccine. When this participant was asked if they had any concerns regarding the safety of the vaccine, their response was:

“*I mean, as far as safety of vaccines, no. Not really. I mean, the main risk that can come is myocarditis. Is there a small risk of myocarditis in adolescent boys? Yes. When you have that risk benefit discussion, if you actually look at the numbers, and there’s a lot of great visualizations of the numbers, it’s not even a comparison. The risk, if you get COVID, you are many more times likely to get myocarditis, and so you are preventing that. It’s kinda of like, is there a risk of wearing a seatbelt? Yeah. I see people in the emergency department with broken ribs from a seatbelt, or liver injuries from a seatbelt, but for every one of those I see 1,000 more that these lives were saved by a seatbelt.”*

To overcome child vaccine hesitancy, participants explained how children can expose family members or friends who are at higher risk for severe complications from COVID. Just because youths may not be as likely to develop severe complications from COVID, that does not mean they are any less likely to spread the virus to more vulnerable population if unvaccinated. It became evident for our health officials that everyone who can be vaccinated should have the chance to receive the vaccine. One health official even admitted they were unsure of the safety and effectiveness of the vaccine when it was initially rolled out, but they continuously saw the COVID death reports and hospitalizations statistics, so they trusted in science and promoted the vaccine. The challenges health officials encountered while weighing resources when promoting the vaccine among groups when vaccine safety concerns are included as the final branch in the flowchart below (Figure 1). This public health official referred to the vaccine as a gamble but viewed the long-term effects of COVID and the ability to contract COVID more than once as being a greater risk than the vaccine. With the vaccine being created at record speed and its safety being a topic of debate, public health officials had to strategically emphasize how the risks associated with the complications of COVID, ability to contract COVID multiple times, transmitting the virus to high risk loved ones, and the unknown long-term effects of COVID outweighed the risks associated with the COVID vaccine.

## Discussion

### Effects of political polarization

This study investigated how Milwaukee County public health officials navigated political polarization of the COVID vaccine and misinformation in their communities. We identified three themes constructed from recurring observations and strategies. Factors similar to those found to influence vaccine uptake were mentioned in our participants’ responses with particular emphasis on misinformation and political polarization of the vaccine creating challenges for promoting the vaccine in their communities. Rapid, vast dissemination of misinformation in media, political polarization of COVID and the vaccine, and risk assessment of disease severity vs. vaccine safety have received research attention as barriers to vaccination for COVID at both a collective and individual level. Research suggests populations across the world who believe misinformation about the vaccine and severity of COVID have increased vaccine hesitancy; for instance, voters affiliated with the Republican Party have higher rates of vaccine hesitancy than Democrats (22). Effects from political decisions regarding COVID prevention measures can be seen across the different states. States with Republican leadership saw fewer adoptions of COVID prevention recommendations with more delays and increased mortality across races than states with Democratic leadership (23). Wisconsin is one of the most divided states in the nation as noted by its election results. Milwaukee County is not quite as divided as the state with two-thirds of voters in favor of the Democratic presidential candidate, but when voting for their congressional representative, two-thirds of votes were for the Republican candidate (24).

### Combatting misinformation

Social media is used more than ever for disseminating news; however, it can lead to rapid spread of misinformation and lead to increasing vaccine hesitancy among communities (11, 25). This study provides evidence to suggest public health officials felt they were not trusted and lacked support when enacting pandemic prevention guidelines and promoting the COVID vaccine. The abundance of contradictory and misinformative messages, often through social media, challenged the actions of our public health officials making it more difficult for them to protect their communities from COVID. The high volume and reach of misinformative posts on social media networks has been explored through various studies (26). Public health officials described the different strategies they used to navigate the challenges that they faced when promoting the vaccine and safety recommendations all while struggling with increasing burnout and high employee turnover. Our public health officials faced a combination of challenges when trying to weigh their resources while attempting to promote the COVID vaccine. The limited supply of the COVID vaccine early-on during the pandemic, in combination with the limited personnel and funds of health departments made for difficult decisions when promoting the vaccine among certain populations who were considered to be less at risk for severe COVID complications who could still contract and spread COVID. These individuals were found to be less likely to accept the vaccine according to our health officials, which may have been due to the early on promotion of the vaccine for more at-risk groups. This helped create a false sense of security for less at-risk groups as they may have felt they did not need to be vaccinated. Once the US had a stable supply of the vaccine, the challenges for promoting the vaccine among less at-risk groups only grew. COVID fatigue began to set in for many people and some of those who had abstained from being vaccinated as they felt they did not need it as much as other more at-risk people, had no desire to receive it after it became available to everyone. Efforts to vaccinate everyone are still underway, but as more people weigh the decision to vaccinate and health departments are forced to weigh their resources, the trajectory for future booster vaccination coverage is ambiguous. It is important we learn from the early-on COVID vaccine promotion strategies and enforce the need for a highly vaccinated population to keep a virus from spreading and evolving.

### Application of theory

Findings from this study can be used to guide interventions to promote vaccine uptake. Future research is needed to understand the perspectives of vaccine-hesitant individuals to learn more about the beliefs that drive the decision to vaccinate or not. Misconceptions and a general lack of trust in vaccines can be assessed, accounted for, and evaluated by using health communication strategies, such as the Health Belief Model (27). The Health Belief Model (HBM) serves as the framework for many public health campaigns. The HBM uses six constructs to predict health behavior: risk susceptibility, risk severity, benefits to action, barriers to action, self-efficacy, and cues to action (28). Components of this theory were intertwined in the vaccine promotion strategies our public health officials used as they focused on disseminating accurate information about the severity of COVID complications, often underestimated by the public, and the benefits of vaccination. The Theory of Planned Behavior (TPB) is the theoretical framework for the investigation of the influences on a person’s decision to vaccinate as it allows us to better understand why something or someone else affects a person’s decision making. The TPB states that behavioral intention is determined by more positive attitudes toward the behavior, approval of significant others for the behavior (subjective norms), and a sense of personal control over the behavior (perceived behavioral control) (29). Public health officials reported how they believed the rapid circulation of misinformation and political polarization of COVID influenced individuals’ decisions to vaccinate. Strategies they implemented had to overcome these influences by targeting components of TPB. Public health officials incorporated TPB and targeted negative influences by developing grassroot campaigns, promoting community leadership and empowerment as they found community members were just as heavily influenced, if not more so by those around them in their own community who had their best interests in mind. Public health officials were not trusted and supported as well as they should have been due to the controversial, politically polarized misinformation regarding COVID circulating through communities who believed misinformation from sources they found to be more trusted or favorable than accurately informative public health officials.

### Vaccine promotion recommendations

Health officials had to develop new plans to promote COVID safety recommendations and awareness among the public, all while counteracting circulating misinformation and politically polarizing media sources. To do this, many public health organizations turned to social media platforms. Research suggests social media campaigns can successfully inform the public on accurate COVID information to increase public awareness and education so that behavioral change can occur (30). Social media became a frontier for COVID information dissemination requiring health officials to learn more about effective social media campaign strategies and navigation of various social media platforms that they may have had little experience with prior to the pandemic. To combat misinformation spreading through communities who may not have viewed public health departments as the primary source of COVID prevention information, several public health officials enacted grassroots campaigns and tailored social media messages to increase vaccine acceptance and reduce misinformation that might be causing vaccine hesitancies. The public health officials created their strategies knowing that individuals were more perceptive to messages delivered by people they trust. The Theory of Planned Behavior also suggests that mass dissemination of accurate information can be more effective when tailored for a specific audience who will then reshare the information, as seen with multiple social media platforms used by public health officials.

### Future implications

The climate of public health changed drastically once the pandemic began, but it also brought forth long standing issues about how the public health system is supported. Public health officials faced extreme challenges as a divided political system failed to properly fund health departments and support their evidence-based guidelines, restrictions, and recommendations (31). Even prior to 2019, governing parties had not properly supported public health systems in the US. The pandemic proved just how chronically underfunded and underserved the public health system was. Since 2008, the public health workforce has decreased by over 20% while 62% of local health departments have had no increases in funding (32). During the pandemic, public health officials across the nation, including several of our interview participants, were forced to change their roles and/or responsibilities to help with pandemic prevention, monitoring, and mitigation. Officials who had no experience with emerging infectious disease projects had to stop working on their projects to help with pandemic related projects. Opioid abuse prevention, blood lead investigations, health inspections, and countless other projects were halted as health departments did not have the funding or staff to keep up with the COVID response and these other areas at the same time (32). Health departments did not fare well when employees had to switch from their preferred projects to work on COVID related projects. Many of the health officials in our study noted how the reorganization of their department and changing of roles led to the resignation of many public health officials. The strenuous conditions public health officials faced began long before the pandemic. Moving forward, it is essential that our nation focuses on developing, funding, and supporting our public health system. Public health officials are experts in their field. Their expertise should be recognized and supported by political and administrative leaders who make decisions regarding mandates and guidelines created to better protect the health of public.

Additionally, the concerns expressed by public health officials regarding burnout and lack of support should be used to improve the emergency preparedness system in Milwaukee County and perhaps in other communities. This study found many public health officials were overwhelmed from the start of the pandemic, highlighting the lack of experienced personnel, support from the political system and administrative organizations, and communication with decision making entities. Experts predict future disease outbreaks will occur, and another pandemic is imminent. If another were to occur, it is essential we learn from the past 3 years by providing more support to our public health system, so they can better serve and protect members of the community without the backlash they faced during the COVID pandemic.

### Limitations

This study could have benefited from asking public health officials additional interview questions addressing what theoretical models (if any) they used in crafting their messages, such as, using concepts from the Health Belief Model to understand vaccine decision making of community members. We were able to draw inferences from the interview results, but more precise questions would lead to more precise conclusions. Moving forward this study could expand outside of Milwaukee County to the greater Wisconsin area, adding more public health officials to our study who can provide insight into perhaps more conservative counties of Wisconsin.

## Conclusions and implications

Through interviews with seven Milwaukee County public health officials and qualitative, thematic analysis, this study successfully identified factors contributing to COVID vaccine acceptance in Milwaukee County, factors affecting public health decision making during the pandemic, and the strategies public health officials used to promote the vaccine and enforce COVID safety precautions. Misinformation in the media, political polarization of COVID and its contribution to burnout among public health workers, and the weighing of COVID severity versus limited vaccine promotion resources created challenges for public health officials in Milwaukee throughout the pandemic. Public health officials guided much of the COVID pandemic response and the initial vaccine rollout. Many times, they received little support from political leaders as the vaccine became politically polarized and they were required to develop strategies to overcome an array of circulating myths and misinformation about the vaccine. By implementing tailored responses to the challenges that they faced, public health officials were able to create strategies for increasing vaccine acceptance and reducing hesitancies. Moving forward, public health officials need the support of all leaders (political, administrative, and community) to be able to best serve their community.

## Disclosures

The project was approved by the Medical College of Wisconsin Institutional Review Board.

## Data availability statement

The original contributions presented in the study are included in the article/supplementary material, further inquiries can be directed to the corresponding authors.

## Author contributions

All authors listed have made a substantial, direct, and intellectual contribution to the work and approved it for publication.
